# An update on chronic complications of diabetes mellitus: from molecular mechanisms to therapeutic strategies with a focus on metabolic memory

**DOI:** 10.1186/s10020-024-00824-9

**Published:** 2024-05-26

**Authors:** Tongyue Yang, Feng Qi, Feng Guo, Mingwei Shao, Yi Song, Gaofei Ren, Zhao Linlin, Guijun Qin, Yanyan Zhao

**Affiliations:** 1 Division of Endocrinology, Department of Internal Medicine, The First Affiliated Hospital of Zhengzhou University, Zhengzhou, 450052 China; 2 Traditional Chinese Medicine Integrated Department of Nephrology, The First Affiliated Hospital of Zhengzhou University, Zhengzhou, 450052 China; 3 Research Institute of Nephrology, Zhengzhou University, The First Affiliated Hospital of Zhengzhou University, Zhengzhou, 450052 People’s Republic of China

**Keywords:** Metabolic memory, Diabetic complications, Molecular mechanisms, Models of metabolic memory, Treatment progress

## Abstract

Diabetes mellitus, a chronic metabolic disease, often leads to numerous chronic complications, significantly contributing to global morbidity and mortality rates. High glucose levels trigger epigenetic modifications linked to pathophysiological processes like inflammation, immunity, oxidative stress, mitochondrial dysfunction, senescence and various kinds of cell death. Despite glycemic control, transient hyperglycemia can persistently harm organs, tissues, and cells, a latent effect termed "metabolic memory" that contributes to chronic diabetic complications. Understanding metabolic memory's mechanisms could offer a new approach to mitigating these complications. However, key molecules and networks underlying metabolic memory remain incompletely understood. This review traces the history of metabolic memory research, highlights its key features, discusses recent molecules involved in its mechanisms, and summarizes confirmed and potential therapeutic compounds. Additionally, we outline in vitro and in vivo models of metabolic memory. We hope this work will inform future research on metabolic memory's regulatory mechanisms and facilitate the development of effective therapeutic compounds to prevent diabetic complications.

## Introduction

Diabetes mellitus (DM) is a chronic metabolic disease characterized by elevated blood glucose caused by deficiency or resistance to insulin (Joslin 1946). Chronic hyperglycemia can lead to multiple organ injury, thereby causing various complications, such as diabetic retinopathy (DR), diabetic kidney disease (DKD), and diabetic cardiovascular disorders (Zheng et al. 2018). Epidemiological studies have revealed that DM has emerged as a significant threat to human mortality. At present, the International Diabetes Federation (IDF) estimates that DM affects approximately 536.6 million adults worldwide, and that number is expected to increase to 783.2 million by 2045 (Sun et al. 2022).

In addition to its high incidence, the pathogenesis of diabetic complications is also very complex. In the early stages of DM, hyperglycemia induces oxidative stress and excessive advanced glycation end product (AGE) formation (Domingueti et al. 2016). As the disease progresses, protein glycation and mitochondrial DNA (mtDNA) damage to respiratory chain components can in turn exacerbate oxidative stress injury (Bhatti et al. 2022). Metabolic imbalance then promotes inflammation through binding receptors for glycation products to cause senescence or cell death (Takahashi et al. 2022; Phoenix et al. 2022; Teodoro et al. 2018). These structural changes can lead to various diabetes-related vascular complications (Teodoro et al. 2018). To improve the mechanisms described above, multiple novel hypoglycemic agents, such as sodium glucose co-transporter 2 inhibitor (SGLT2i), dipeptidyl peptidase 4 inhibitors (DPP4i) and glucagon-like peptide 1 receptor agonists (GLP-1RAs), have been applied in clinical practice (Mouhayyar et al. 2020; Nathan et al. 2013; Zhang and Wu 2014) (Mostafa et al. 2016; Mostafa et al. 2015). However, early hyperglycemia can still lead to a variety of diabetic complications. Fortunately, the novel concept of “metabolic memory” may explain this phenomenon. Metabolic memory, also known as hyperglycemic memory, arises from the enduring presence of an underlying driver. The persistence of cellular changes and characteristics represents the organism's recovery of a prior metabolic state, potentially playing a pivotal role in the etiology of DM and its chronic complications (Reddy et al. 2015).

In this comprehensive review, we aim to delve into the research chronology and distinct characteristics of metabolic memory. Additionally, we present a summary of the diverse molecular mechanisms that govern its regulation. By emphasizing its prevalence and profound implications, we highlight the significance of metabolic memory in various chronic diabetes complications. Furthermore, we delve into potential mechanisms and pharmacological advancements related to metabolic memory. Additionally, we consolidate information on various in vitro and in vivo models of metabolic memory. We hope that this review can offer valuable insights into the intricacies of metabolic memory, thereby paving the way for novel therapeutic strategies for the treatment of DM and its complications.

## Overview of metabolic memory

**Fig. 1:**
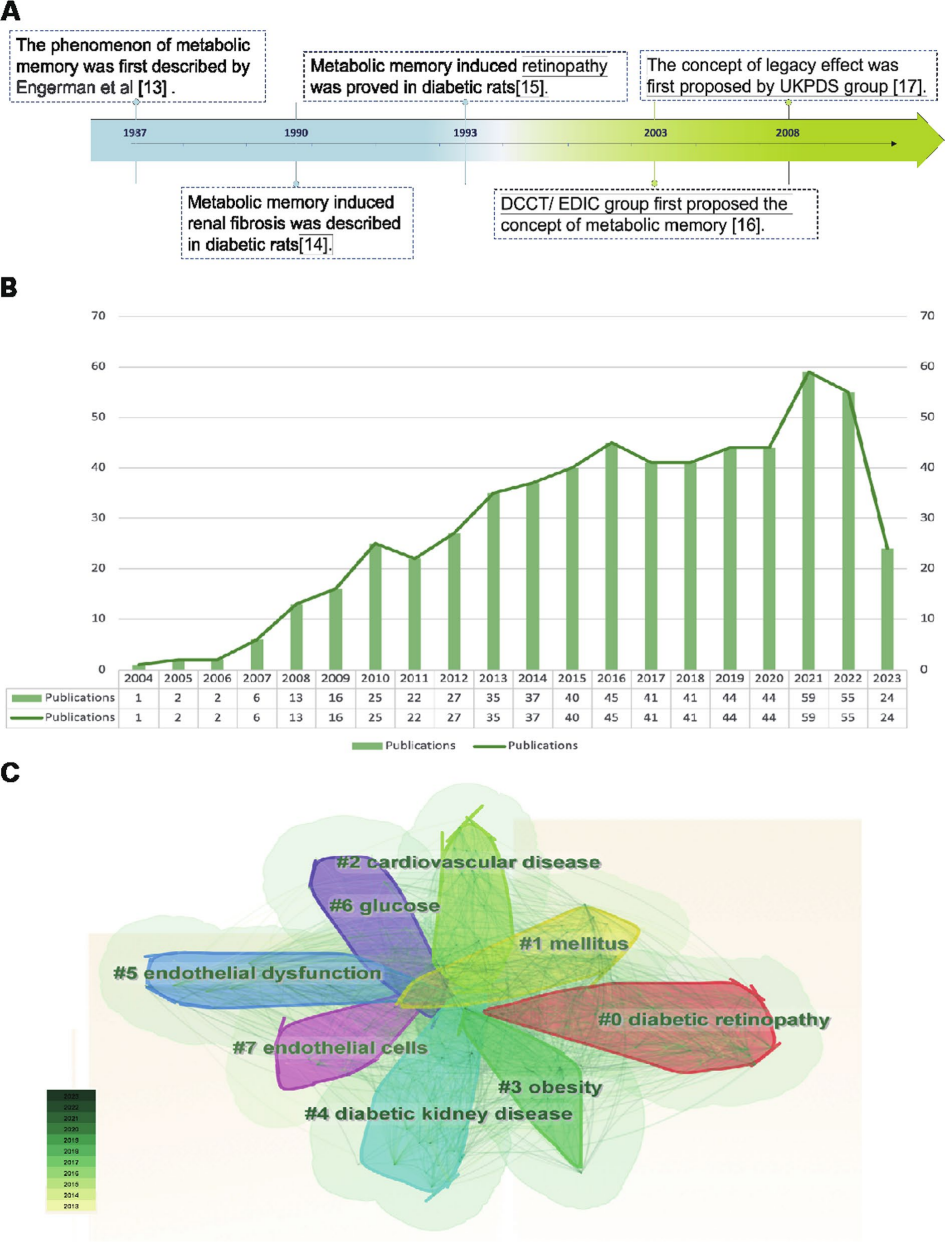
Overview of metabolic memory. **A** Chronological depiction of key events in the development of metabolic memory. **B**, **C** Bibliometric analysis exploring the intersection of metabolic memory and diabetic complications. Search criteria were set as follows: TS = ((“metabolic memory” OR “hyperglycemic memory”) AND (“diabetes” OR “diabetic”)) with a date range of DOP = (2013–08-01/2023–08-01). **B** Illustration of the annual trend in the number of published articles. **C** Clustered view of the key terms and concepts emerging from the literature

The metabolic memory of diabetes refers to the observation that patients are vulnerable to developing diabetic complications due to early hyperglycemia, even if effective hypoglycemic agents are taken to maintain blood glucose within normal levels in the later stage of DM. As shown in Fig. 1A, in 1987, Engerman et al. (Engerman and Kern 1987) first described the phenomenon of metabolic memory that decreased hyperglycemia to normal levels after 2.5 years of exposure in diabetic dogs, and the incidence of DR was still high (Engerman and Kern 1987). In addition, high glucose caused an increase in fibronectin and collagen IV expression that could not be reversed even after restoration to normal levels in diabetic rats in 1990 (Roy et al. 1990). In 1993, Hammes et al. (Hammes et al. 1993) further described the exposure time more accurately. Their research indicated that islet transplantation to diabetic rats could prevent the occurrence of DR within 6 weeks after onset. However, at 12 weeks after onset, DR still occurred. Later, in 2003, the Diabetes Control and Complications Trial (DCCT) with further follow-up in the Epidemiology of Diabetes Interventions and Complications (EDIC) study (DCCT/EDIC), where the concept of "metabolic memory" was first proposed, demonstrated that initial hyperglycemia still increased the risk of long-term diabetic complications, although the HbA1c of the intensive treatment group and conventional treatment group was maintained at similar levels (Writing Team 2003). In 2008, the United Kingdom Prospective Diabetes Study (UKPDS) again demonstrated the term “legacy effect”, in which early intensive glucose lowering can lead to long-term benefits in patients with newly diagnosed type 2 diabetes (Holman et al. 2008; Ranjit Unnikrishnan et al. 2011). Both "metabolic memory" and the “legacy effect” refer to the long-term effects of blood glucose on macrovascular and microvascular complications of diabetes. However, the concept of metabolic memory may focus on the negative effect of hyperglycemia impairment, while the legacy effect mainly focuses on the positive influence of effective treatments.

The bibliometric analysis of the research published on metabolic memory in the decade following its formal designation in 2004. Based on the information provided by the Web of Science (webofscience.com), we analyzed the scientific output related to metabolic memory and diabetes from 2000 to 2022. In total, 579 articles were identified. The trend of research related to metabolic memory and diabetes is displayed in Fig. 1B, which shows a steady upward trend since its official naming in 2004, particularly in 2021–2022. Cluster analysis of high-frequency keywords related to metabolic memory and diabetes was performed using CiteSpace (Fig. 1C). The clustering outcomes revealed a preponderance of research centering on the interplay between metabolic memory and diabetes, with a focus on DR, cardiovascular disease, endothelial dysfunction, DKD and obesity. Notably, obesity is intricately intertwined with glycemic and metabolic homeostasis, as evident in previous studies (El-Mesallamy et al. 2013; Aboouf et al. 2015; Khella et al. 2017). However, Zapata et al. (2022) also observed that obesity elicits a persistent metabolic imprint that persists despite weight loss, phenotypically resembling metabolic memory. Despite this, the existing literature consistently associates metabolic memory with glycemic fluctuations. This preponderance of findings can be partially attributed to the inherent constraints of bibliometric analysis, including the challenges associated with the precision and breadth of bibliographic databases, the absence of contextual understanding, and potential biases towards high-impact journals or specific research domains. Consequently, there is a pressing need for further exploration in this realm to clarify the intricate relationships among obesity, metabolic memory, and glycemic fluctuations. Our review primarily centered on metabolic memory and the potential long-term health implications of transient abnormalities in glucose metabolism.

## The main molecular mechanisms of metabolic memory

**Fig. 2:**
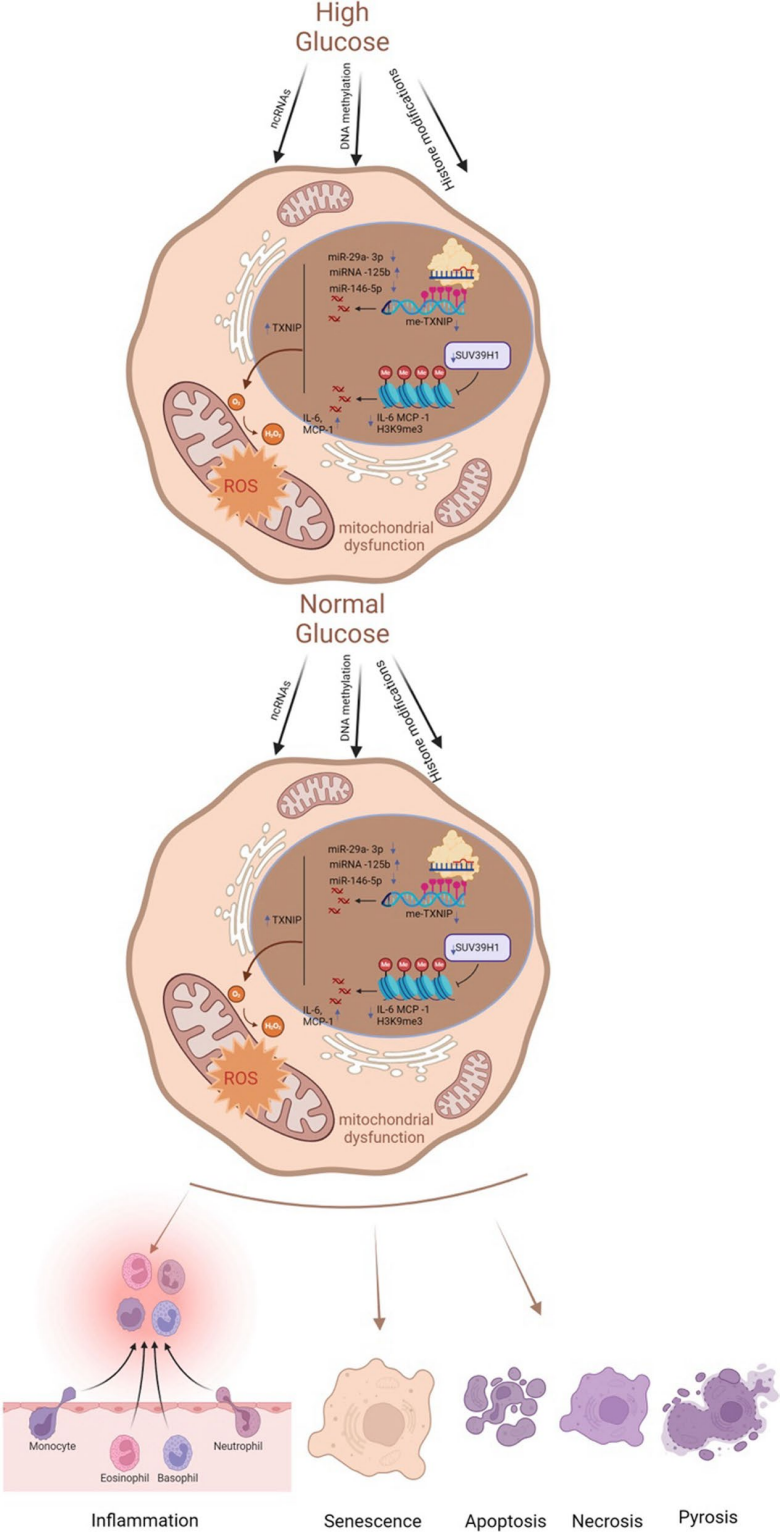
Key molecular mechanisms of metabolic memory. Despite the normalization of glucose levels, epigenetic modifications, inflammatory and immune responses, oxidative stress, mitochondrial dysfunction, cellular senescence, and apoptosis persist. These processes constitute the core molecular mechanisms underlying metabolic memory. *ncRNAs* noncoding RNAs, *TXNIP* thioredoxin-interacting protein, *me-TXNIP* thioredoxin-interacting protein, *IL-6* interleukin-6, *MCP-1* monocyte chemotactic protein 1, *H3K9me3* trimethylated histone H3 at lysine 9, *ROS* reactive oxygen species

The underlying mechanisms of metabolic memory and diabetic complications include inflammation and immunity, oxidative stress and mitochondrial dysfunction, senescence and various kinds of cell death. In fact, these mechanisms involve crosstalk with each other (Galicia-Garcia, et al. 2020; Berezin 2016). Epigenetic modifications can lead to inflammation, oxidative stress, and senescence, which in turn can be regulated by these mechanisms (Fig. 2).

## Epigenetic mechanisms involved in metabolic memory

Epigenetic mechanisms, including DNA methylation, histone modifications and noncoding RNAs (ncRNAs), can influence transcription activity and the generation of a heritable phenotype without changing DNA sequences (Goldberg et al. 2007). Emerging studies have indicated a key role for epigenetic modifications in the regulation of physiological and pathological processes associated with diabetic complications and metabolic memory (Chen and Natarajan 2022). Thus, this section mainly focuses on various modifications involved in hyperglycemic memory.

### DNA methylation

DNA methylation, the most stable and widely reported epigenetic mechanism, is considered the primary transcriptional regulator. To investigate the relationship between hyperglycemic memory and DNA methylation, Chen et al. (2016) selected patients with type 1 diabetes mellitus (T1DM) from DCCT and EDIC studies. They discovered twelve distinctively annotated differentially methylated loci that exhibited a strong association with hyperglycemia and were intricately linked to diabetic complications. Notably, among these loci, thioredoxin-interacting protein (*TXNIP*) is a pivotal gene in the pathogenesis of diabetic complications. Transient hyperglycemic episodes were found to trigger hypomethylation at the 3’ untranslated region (3′ UTR) of TXNIP, leading to persistently elevated expression of this protein in peripheral blood cells (Thielen and Shalev 2018). This, in turn, triggered oxidative stress and triggered apoptotic and pyroptotic processes (Choi and Park 2023). Moreover, Park et al. (2014) derived foot fibroblasts from patients with diabetes with or without ulcers and from nondiabetic subjects without foot ulcers. Then, foot fibroblasts from patients with DM were cultured for four passages under normoglycemic conditions, and global and genome-wide DNA methylation profiles were used to identify alterations in DNA methylation. Their results illustrated that DNA methylation and metabolic memory were associated with poor wound healing outcomes in patients with diabetic foot ulceration. Similarly, proximal tubular epithelial cells (PTECs) derived from patients with or without diabetes were cultured via normoglycemic culture for four passages. After integrative omics analysis, multiple changes in DNA methylation sites were detected; among these changes, *HNF4A* may regulate epigenetic and hyperglycemic memory in DKD (Bansal et al. 2020).

In summary, these studies suggest that DNA methylation plays a vital role in metabolic memory and diabetic complications. In addition, as DNA methylation is involved in hyperglycemic memory, a review speculated that emerging m6A RNA methylation may also be a potential mechanism (Kumari et al. 2021). However, this theory remains to be confirmed in the future.

### Histone modifications

Histones, including the corehistones H2A, H2B, H3, and H4 and the linker histone H1, can bind tightly to DNA to form nucleosome structures. Histone posttranslational modifications (HPTMs) refer to covalent modifications in which different modifications are added to one or several amino acid residues on the tails of histones. The modified histones change the loose or tight binding state between histones and DNA to effectively regulate gene transcription. The most common HPTMs are acetylation (Kac) and methylation (Kme) (Jin and Jeong 2023; Sun et al. 2023). Filgueiras et al. (2017) demonstrated that STAT1/MyD88 mRNA and protein levels remained elevated for a minimum of six days in macrophages from diabetic mice. This upregulation could be attenuated by the histone acetyltransferase (HAT) inhibitor anacardic acid. Furthermore, in the skeletal muscle tissue of diabetic mice, persistent enhanced *Ped/Pea-15* expression was related to histone H3 lysine 4 monomethylation (H3K4me1) but not histone H3 Lys27 acetylation (H3K27Ac). The high expression of H3K4me1 remained stable even after re-exposure to 5 mM glucose-containing medium. However, there was a prompt loss of acetylation at K27 on histone H3 and a reduction in p300 recruitment at *Ped/Pea-15* (Vastolo et al. 2018). In addition to H3K4me1, H3K9me3, a crucial repressive and relatively stable epigenetic chromatin mark, also contributes to metabolic memory in vascular smooth muscle cells (VSMCs) derived from db/db mice. The persistent downregulation of H3K9me3 and the inflammatory phenotype could be reversed by overexpressing suppressor of variegation 3–9 homolog 1 (Suv39h1), which is a histone methyltransferase (Sun et al. 2023). H3K4me1 and H3K9me3 also regulate metabolic memory in CMs and vascular endothelial cells, respectively (Yu et al. 2012; Okabe et al. 2012; Mao et al. 2019). Regrettably, relatively few studies on HPTMs and metabolic memory, especially some emerging HPTMs, such as lactylation, ubiquitination and glycosylation.

### ncRNAs

ncRNAs, which mainly include microRNAs (miRNAs) and long noncoding RNAs (lncRNAs), play a vital role in diabetes and its complications as well as multidrug resistance (Li et al. 2022a; Mahmoud et al. 2021). As another major mechanism of epigenetic regulation, ncRNAs can regulate gene expression by modulating protein synthesis at the posttranscriptional and translational levels (Taft et al. 2010). miRNAs, a class of endogenous single-stranded RNAs composed of 20–22 nucleotides, can participate in regulating posttranscriptional gene expression by binding to target mRNAs (Krol et al. 2010). Currently, various miRNAs have been reported to participate in metabolic memory and diabetic complications. To identify hyperglycemic memory-related miRNAs in human aortic endothelial cells, Zhong et al. (2015) used a miRCURY LNA array to screen for transcriptional changes in the normal glucose, high glucose and metabolic memory groups. After validation in vitro and in vivo, *miR-125b, miR-29a-3p, and miR-146a-5p* were shown to potentially be important for metabolic memory. Notably, *miR-125b* was the only miRNA confirmed to be related to metabolic memory, specifically targeting Suv39h1 to promote inflammation in VSMCs from diabetic mice (Villeneuve et al. 2010). Subsequently, Costantino et al. (2016) screened 268 miRNAs that remained significantly altered after 3 weeks of intensive glycemic control with insulin from heart samples. The majority of miRNAs related to metabolic memory effects, according to an ingenuity pathway analysis, regulate the myocardial pathways of apoptosis, autophagy, oxidative stress, fibrosis, hypertrophy and heart failure. Regrettably, they verified miRNA expression in left ventricular samples from controls, diabetic mice, and diabetic mice treated with insulin without further exploring the underlying mechanisms involved. In addition, *miR-23b-3p* has been proven to regulate high glucose-induced metabolic memory via the SIRT1-dependent signaling pathway in DR (Zhao et al. 2016). However, in-depth studies of the links between key lncRNAs and the crosstalk between lncRNAs and miRNAs in metabolic memory and diabetic complications still need further exploration.

## Inflammation, immunity, oxidative stress and mitochondrial dysfunction

High blood glucose can induce chronic metabolic inflammation, which contributes to the development of various complications (Nedosugova et al. 2022). Monocytes and macrophages, crucial components of immunity, participate in inflammation in diabetic complications. The proinflammatory activation of macrophages within the liver and adipose tissue can initiate the recruitment and promotion of macrophage polarization, thereby inducing these cells to secrete inflammatory cytokines, including IL-1β, IL-6, and TNF-α. This, in turn, results in immune imbalance, highlighting the critical role of macrophage activation in the pathogenesis of inflammatory conditions (Bleriot et al. 2023; Ding et al. 2022). To further investigate the intricate relationships among inflammation, immunity, and metabolic memory, Mossel et al. (2020) investigated metabolic memory in primary human macrophages. Their findings revealed that even after normalizing glucose levels, the expression of *S100A9* and *S100A12* remained elevated, potentially due to transient hyperglycemia-induced histone methylation at the promoters of these genes. In addition, innate immune cells, which are integral to diabetes-related complications, can establish nonspecific immunological memory (trained immunity) through epigenetic regulation. Thiem et al. (2021) established both in vitro and in vivo trained immunity models using bone marrow cell transplantation and monocyte isolation. Their study demonstrated that glucose modulation of innate immune cell histone methylation levels can persist, leading to increased glycolysis and exacerbated inflammatory responses even after glucose normalization. Given these insights, diabetes and its complications related to oxidative stress and inflammation, as well as immunity, can significantly benefit from vitamin E intake (Hamdy et al. 2009).

In addition to the aforementioned factors, oxidative stress and mitochondrial dysfunction play essential roles in metabolic memory (Peng, et al. 2020). An imbalance between oxidative and antioxidative processes gives rise to oxidative stress, which can trigger lipid accumulation, inflammation, and fibrosis in diabetic complications (Zhang et al. 2020). Reactive oxygen species (ROS), a hallmark of oxidative stress, encompass a range of free radicals, including superoxide anions, hydroxyl and peroxyl radicals, and other compounds capable of generating free radicals (Halliwell 2006). Since mitochondria are key intracellular sources of ROS, mitochondrial dysfunction is intimately linked to oxidative stress (Cojocaru, et al. 2023). Multiple studies have established that oxidative stress and mitochondrial dysfunction are integral to the mechanism of metabolic memory in diabetic complications, particularly in the progression of DR (Wang et al. 2018; Zhong and Kowluru 2013; Voronova et al. 2017; Drzewoski et al. 2009). Sirtuin-1 (SIRT-1) functions as a modulator of antioxidant defense, energy metabolism, and organelle homeostasis, making it a key player in oxidative stress and mitochondrial dysfunction in various diseases (Kung, et al. 2021; Li et al. 2022b). Lee et al. (2022) demonstrated that SIRT-1 was a link between hyperglycemic memory and oxidative stress and mitochondrial dysfunction in DR. Additionally, Kowluru et al. (2023) provided evidence that transient hyperglycemia results in a persistent imbalance in mitochondrial fission, mitophagy, and new mitochondrial formation, ultimately leading to oxidative stress in DR. Beyond mitochondrial dysfunction, oxidative stress intersects with other organelle dysfunctions, including endoplasmic reticulum (ER) stress, Golgi apparatus stress, and lysosomal homeostasis (Maamoun et al. 2019; Gong et al. 2022; Jiang et al. 2011). However, the intricate relationships between these processes remain largely unexplored and require further investigation.

## Senescence and cell death

Cellular senescence, a type of permanent proliferative arrest without cell death, is divided into epigenetically induced senescence, oxidative stress-induced senescence and DNA damage-induced senescence (Hernandez-Segura et al. 2018). The process of senescence is closely related to programmed cell death (PCD) (Galluzzi and Myint 2023). When cellular damage cannot be efficiently repaired, irreversible dysfunction of cells can lead to PCD, including apoptosis, autophagy, pyroptosis and ferroptosis (Moujalled et al. 2021). Recently, a *p21*-dependent pathway was identified that contributes to senescence and hyperglycemic memory in DKD (Al-Dabet et al. 2022). Furthermore, Mansour et al. (2023) demonstrated that overexpressed *p21* can lead to senescence and increase the expression of BAX, a pro-apoptotic gene, to alleviate apoptosis. These results indirectly illustrate that p21, a key gene in metabolic memory, also participates in senescence and apoptosis and may be a promising target. Moreover, in DR, temporary high glucose could lead to consistent upregulation of miR-195 to decrease the expression of its target gene *Bcl-2*, which is an antiapoptotic gene (Liu et al. 2019a). This research suggested that epigenetic mechanisms, as representative ncRNAs, may interact with senescence and cell death in hyperglycemic memory. Nevertheless, how do other types of cell death regulate metabolic memory in diabetic complications? This question is still unanswered.

## Metabolic memory and chronic complications of DM

**Fig. 3:**
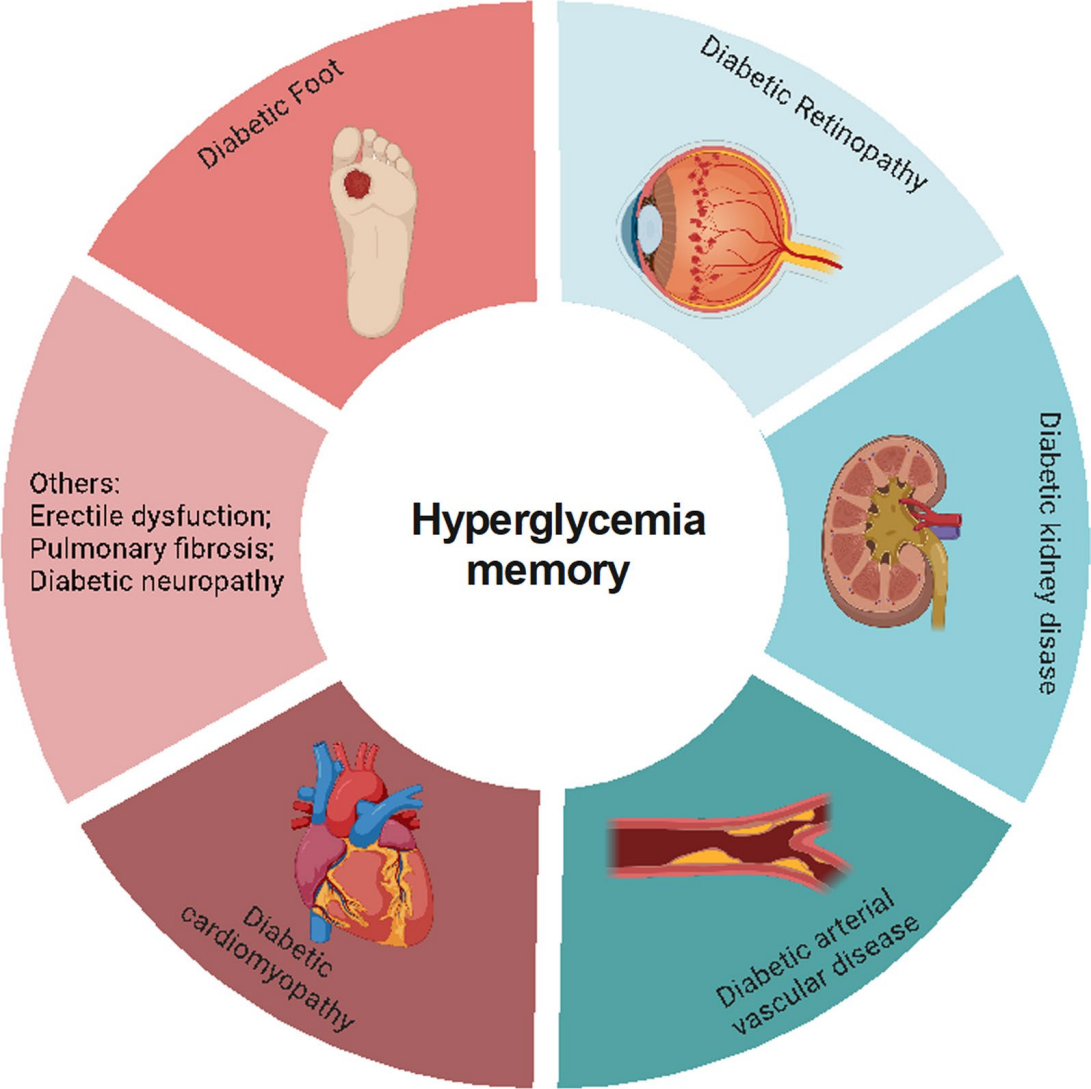
Metabolic memory and chronic complications of diabetes. Hyperglycemia can trigger a range of diabetic complications, including diabetic cardiomyopathy, diabetic arterial vascular disease, diabetic kidney disease, diabetic retinopathy, and diabetic foot. This figure illustrates the intricate relationship between metabolic memory and these chronic conditions

Multiple large-scale clinical trials have verified that early intensive glycemic control can reduce the incidence and progression of macrovascular and microvascular complications of diabetes, including diabetic cardiovascular disorders, DKD, DR, and diabetic foot disease (DF) (C., I. 2003; Cuore et al. 2023; Brown et al. 2010; Nathan et al. 2014; Aiello et al. 2014), which is basically consistent with the results of our bibliometric analysis (Fig. 1C). Numerous studies have also used experiments to elucidate the mechanisms underlying this clinical phenomenon in diabetic complications (Yamagishi et al. 2017; Zhong et al. 2023; Kato and Natarajan 2019). Thus, in this section, we will discuss the relationship between metabolic memory and chronic complications of DM (Fig. 3).

## Diabetic cardiovascular disorders and metabolic memory

Diabetic cardiovascular disorders, including diabetic cardiomyopathy (DCM) and arterial vasculopathy, are the leading causes of death among patients with diabetes (Fang et al. 2004). Elevated blood glucose stimulates inflammation, regulates immune cells, and promotes the production of cytotoxic free radicals, thereby attacking myocardial cells and vascular endothelial cells (Johnson et al. 2022; Xie et al. 2022). Under the action of these mechanisms triggered by high glucose, damaged cells further secrete harmful irritants, which promote the transdifferentiation of other cell types into cardiac fibroblasts (Cheng et al. 2023). Subsequently, various adhesion molecules and adipokines, such as adiponectin, influence these fibroblasts, activating them to migrate and aggregate, thus exacerbating myocardial and vascular injury (El-Mesallamy et al. 2011). However, evidence from clinical trials has indicated that even with intensified blood glucose control, patients with diabetes are still at risk for diabetic cardiovascular diseases due to metabolic memory. This section discusses the relationship between diabetic cardiovascular diseases and metabolic memory.

### DCM and metabolic memory

DCM is a cardiovascular complication that arises from DM and causes alterations in cardiac structure and function, independent of hypertension, coronary atherosclerotic heart disease, or any other known cardiac risk factors (Jia et al. 2018). Previous studies have established that metabolic dysfunction in cardiomyocytes, myocardial interstitial fibrosis, abnormal calcium transients in cardiomyocytes, and cardiac autonomic neuropathy play pivotal roles in the pathogenesis of DCM (Palomer et al. 2018; Marwick et al. 2018). Roy et al. (1990) demonstrated that *fibronectin* mRNA expression increased even after blood glucose returned to normal in streptozotocin (STZ)-induced diabetic rats. Given the mechanisms and manifestations of DCM, metabolic memory may play a key role in its development and progression (Zhan et al. 2022). Additionally, previous studies have shown that miR-320 mediates apoptosis in DCM (Su et al. 2020). Moreover, multiple studies have suggested that cluster of differentiation 36 (CD36) regulates free fatty acid uptake in DCM, and CD36-deficient patients and CD36 knockout mice exhibit a significant reduction in the myocardial uptake of long-chain fatty acids (LCFAs) (Zhang et al. 2021). A recent study revealed a connection between these factors, revealing that miR-320 serves as a central ncRNA in metabolic memory and positively interacts with CD36 to alleviate diastolic dysfunction caused by hyperglycemic memory in cardiomyocytes (Zhan et al. 2023). This finding offers novel insights into the pathogenesis of DCM and its molecular functions.

### Diabetic arterial vasculopathy and metabolic memory

Elevated blood glucose can inflict substantial harm on both the microvascular and macrovascular systems, ultimately leading to endothelial dysfunction, atherosclerosis, and various vascular complications (Li et al. 2023). Observations from studies such as the EDIC and UKPDS revealed that individuals in the intensive treatment group developed fewer microvascular and macrovascular diseases (C., I. 2003; Retnakaran et al. 2006). Jax et al. (2010) argued that structural alterations, including perivascular fibrosis of microvessels, can exert a direct impact on upstream arteries, gradually leading to endothelial dysfunction and, subsequently, the development of atherosclerosis. The endothelium, the largest organ of the body, plays a pivotal role in regulating the functionality of blood vessels. Persistent hyperglycemia leads to oxidative stress, inflammation, and abnormal mitochondrial metabolism, all of which contribute to endothelial dysfunction (Wang et al. 2022). Remarkably, even when transient hyperglycemic conditions revert to normal glycemic levels, oxidative stress and inflammatory factors persist within aortic endothelial cells (El-Osta et al. 2008). Damaged endothelial cells lose their functionality and undergo a process known as endothelial-to-mesenchymal transition (EndMT), during which they transform into mesenchymal cells or myofibroblasts, thereby contributing to pathological fibrosis (Xu and Kovacic 2023; Bischoff 2019). Previous research has shown that hyperglycemic memory can also trigger EndMT and fibrosis (Al-Dabet et al. 2022). In this context, miR-27a, a ncRNA closely associated with EndMT and fibrosis, has been further implicated in the NF-κB/miR-27a-3p/NRF2/ROS/TGF-β/EndMT feedback loop, which regulates metabolic memory in endothelial cells (Liu et al. 2019b; Yao et al. 2022). Reddy et al. (2016) demonstrated that the expression of miR-504 remains persistently high in diabetic VSMCs even after several passages of in vitro culture, enhancing ERK1/2 activation and VSMC dysfunction in atherosclerosis and restenosis.

In summary, metabolic memory is intricately linked to oxidative stress, inflammation, and fibrosis and plays a pivotal role in the pathogenesis of DCM and diabetic arterial vasculopathy. The involvement of ncRNAs, such as miR-320 and miR-27a, points to complex regulatory mechanisms underlying these processes. Nevertheless, other miRNAs, such as miR-423, miR-499, and miR-199a, have been implicated in metabolic memory and the diabetic heart, but further investigation is needed to fully elucidate their roles (Costantino et al. 2016).

## DKD and metabolic memory

DKD is one of the most common and severe complications of DM and is also the leading cause of end-stage kidney disease (ESKD) in the general population (Novak et al. 2016; Collins, et al. 2011). The minimal functional unit of the kidney is the nephron, which consists of the glomerulus and renal tubule. Hyperglycemia can cause or exacerbate injuries in both the glomerulus and renal tubule to induce renal dysfunction.

### Glomerular injury and metabolic memory

The glomeruli are composed of glomerular endothelial cells (GECs), mesangial cells, podocytes and parietal epithelial cells. As GECs serve as the primary barrier to exposure to high glucose conditions, they can initiate crosstalk between mesangial cells and podocytes. Hyperglycemia can increase the permeability of GECs, alter the glycocalyx and induce GEC apoptosis (Dou and Jourde-Chiche 2019). On the one hand, damaged GECs regulate the expression and secretion of endothelin-1 (ET-1), nitric oxide (NO), endothelial nitric oxide synthase (eNOS) and VEGF family members, thereby aggravating the dysfunction of other cell types, including mesangial cells and podocytes (Thomas and Ford Versypt 2022; Mahtal et al. 2021; Zou et al. 2019). Conversely, dysfunction in mesangial cells and podocytes can also deleteriously affect GECs through the regulation of VEGF expression (Fu, et al. 2022; Bartlett et al. 2016). This intricate crosstalk among glomerular cells plays a pivotal role in the pathogenesis and progression of glomerular injury. Notably, even after the restoration of normoglycemia, the damage to these cells persists. Li et al. (2022c) demonstrated that *Sirt7* cooperates with ELK1 to participate in metabolic memory and DKD through the modulation of DAPK3 expression and endothelial inflammation both in vitro and in vivo. Similarly, for podocytes, the expression of SHP-1 remains elevated despite the reduction in blood glucose levels achieved by insulin treatment for the last two months in diabetic mice (Lizotte et al. 2016). Additionally, free fatty acids, such as palmitate, contribute significantly to the development of insulin resistance. Thus, Novak et al. (2016) further demonstrated that a high-fat diet or palmitate can alter H3K36me2 and H3K27me3 on the promoter region of the *FOXO1* gene, thereby regulating metabolic memory in podocytes. This comprehensive understanding of the interactions and responses among glomerular cells highlights the complexity and persistence of glomerular injury in patients with diabetes.

### Tubular injury and metabolic memory

The injury of tubular epithelial cells (TECs), which account for the largest proportion of all cell types in the kidney, is an essential link in the pathogenesis of DKD (Vallon and Thomson 2020). On the one hand, hyperglycemia can cause structural alterations in renal tubules, including renal tubule atrophy, tubular cell hypertrophy, thickening of the tubular basement membrane and tubulointerstitial fibrosis (Slyne et al. 2015; Pourghasem et al. 2015). On the other hand, high glucose conditions can also lead to inflammation, programmed cell death, senescence and mitochondrial dysfunction in TECs (Zhou et al. 2023; Shen et al. 2022; Chang et al. 2021). Among them, cellular senescence in TECs is related to epigenetic modifications, which are the core mechanism of metabolic memory (Shen et al. 2022; Tonna et al. 2010). Recent research identified *p21* as a key hyperglycemic memory-related gene that regulates TEC senescence in DKD, and activated protein C (aPC), an enzyme that epigenetically inhibits redox p66Shc, could inhibit *p21* methylation to ameliorate metabolic memory and senescence (Al-Dabet et al. 2022).

In conclusion, metabolic memory is an emerging mechanism in glomerular and tubular injury. Regrettably, studies on the role of metabolic memory in DKD are rare, especially studies on mesangial cells and the crosstalk between different cell types in the kidney. A recent study on the multimodal integration of single nucleus RNA (snRNA-seq) and an assay for transposase-accessible chromatin sequencing (snATAC-seq) in DKD may provide more information on the epigenetic regulation of chromatin accessibility, which could contribute to the long-term expression of DKD and metabolic memory-related genes (Wilson et al. 2022). However, further studies are still needed.

## DR and metabolic memory

DR, characterized as a neurodegenerative and microangiopathic disease, is the major cause of visual impairment in patients with diabetes, accounting for approximately 30 to 40% of cases (Ting et al. 2016; Altmann and Schmidt 2018). Hyperglycemia remains the major factor that contributes to the development and progression of DR (Cheung et al. 2010). The pathophysiological mechanisms underlying DR are complex and include oxidative stress, inflammation, autophagy, cellular dysfunction and cell death. The inflammatory cascades are primarily triggered by oxidative stress. Both inflammation and oxidative stress stimulate retinal autophagy, which leads to cellular dysfunction and cell death in nerve cells, endothelial cells and pericytes. All these factors may interact with each other, ultimately contributing to the development of DR (Wei et al. 2022; Madsen-Bouterse and Kowluru 2008).

Coincidentally, multiple studies have shown that the mechanisms mentioned above regulate hyperglycemic memory to affect DR pathogenesis (Liu et al. 2023). Metabolic memory-induced retinopathy was initially observed in diabetic dogs, which indicated that DR was not improved by good glycemic control (Engerman and Kern 1987). Tewari et al. (2012) reported that despite the restoration of normoglycemia in retinal endothelial cells, hypermethylation of POLG1 promoters did not change, which resulted in mtDNA replication dysfunction. Liu et al. (2019a) demonstrated that *miR-195* remained upregulated in human retinal pigment epithelial cells (RPEs) following three days of culture under high glucose conditions and subsequent normalization to normal glucose levels for another three days, leading to mitochondrial dysfunction-induced apoptosis. Furthermore, Astragalus polysaccharide (APS) attenuated the expression of miR-195 in a dose-dependent manner. Recent studies have demonstrated that the pathogenesis of metabolic memory-induced microvascular dysfunction in DR is regulated by mitochondrial dysfunction, which can be ameliorated by dopamine, mdivi-1 and leflunomide (Lee et al. 2022; Kowluru and Alka 2023; Mohammad and Kowluru 2022). Therefore, mitochondrial dysfunction may be the core mechanism of metabolic memory in DR. Both DKD and DR are microvascular complications of diabetes, and the kidneys and eyes are mitochondria-rich organs. However, studies on the relationship between mitochondrial dysfunction and hyperglycemia in DKD are limited and may be worthy of further research.

Epigenetic modifications also play a vital role in the progression of DR. The high glucose-induced histone 3 lysine 4 (H3K4) hypomethylation status of retinal Sod2 remains persistent even after reversing hyperglycemia (Zhong and Kowluru 2013). Mishra et al. (2014) also proved that as termination of hyperglycemia injury cannot change H3K4 methylation, the binding activity of the transcription factor Nrf2 remains compromised, which leads to oxidative stress. Furthermore, numerous miRNAs also participate in metabolic memory in DR. Apart from miR-195 mentioned above, *miR-23b-3p* regulates the miR-23b-3p/SIRT1/NF-κB feedback loop to maintain metabolic memory in DR (Zhao et al. 2016). Nevertheless, the mechanisms of DNA methylation, lncRNA or other epigenetic modifications are still relatively unknown.

## DF and metabolic memory

DF, a common and severe complication of DM, is a major cause of extremity amputation, and the worldwide prevalence of DF is 6.3% (Zhang et al. 2017; Afonso, et al. 2021). The risk factors involved in the progression of DF are diabetic neuropathy, vascular insufficiency and immunological dysfunction (Noor et al. 2015). As there were no obvious improvements in wound healing even when glycemic control was achieved in patients with DM, metabolic memory may participate in DF (Zhao et al. 2021; Berlanga-Acosta et al. 2023). Del Cuore et al. (2023) used single nucleotide polymorphism (SNP) analysis in a population with diabetic foot disease. Their results indicated that patients with DF showed predominant expression of the VEGF C2578A CC polymorphism and reduced expression of the AC allele. They also found that miR-217-5p and miR-503-5p may be involved in regulating hyperglycemic memory in DF. Inflammation and DNA methylation are involved in metabolic memory, which is also a key mechanism in DR (Acosta et al. 2008; Deng et al. 2023). The genome-wide DNA methylation profiles of foot fibroblasts indicated that the change in DNA methylation was associated with metabolic memory, especially in patients with poor wound healing outcomes of diabetic foot ulceration (Park et al. 2014). Zhao et al. (2021) further demonstrated that transient hyperglycemia upregulated DNA methyltransferase 1 (DNMT1) expression, leading to the persistent hypermethylation of *Ang-1* during subsequent normoglycemia, which induced inflammation and endothelial dysfunction in vitro and in vivo. These findings implied that epigenetic modifications are a hub contributor to metabolic memory in DR, although the present research is still limited.

## Other diabetic complications and metabolic memory

As high blood glucose can injure multiple tissues and organs, hyperglycemic memory is also associated with other chronic complications of DM. Erectile dysfunction (ED) is a common complication of DM, with an approximate prevalence of 35–90% (Malavige and Levy 2009). A retrospective case‒control study showed that early hyperglycemia exposure could have long-term effects on erectile function in patients with DM, which could be sustained even after good glycemic control (Hui et al. 2021). A previous study showed that hyperglycemia could induce endothelial cell injury to cause microvascular leakage in the lung, which can further lead to pulmonary fibrosis (Lee et al. 2022). Jeon et al. (2023) further illustrated that high glucose-induced microvascular leakage and fibrosis in the lung could not be alleviated even after good blood glucose control. Furthermore, the pathophysiological mechanisms of diabetic neuropathy (DN) are epigenetic modifications, inflammation, oxidative stress and mitochondrial dysfunction, which are similar to the mechanisms of metabolic memory (Jankovic, et al. 2021). Thus, a review suggested that metabolic memory may also take part in the development of DN (Jankovic, et al. 2021). Regrettably, directly relevant research is rare, so more solid evidence is needed.

In summary, numerous studies have demonstrated that metabolic memory plays an important role in the progression of multiple chronic complications in patients with DM.

## Potential therapeutic drugs for metabolic memory

Numerous molecular compounds have been proven to act on the key mechanisms of hyperglycemic memory, such as epigenetic modifications, inflammation, and senescence (Table 1). In addition, some molecular compounds may also regulate metabolism, but there is a lack of clear evidence supporting this possibility. Thus, in this section, we summarize the progress of current studies on metabolic memory-related potential drugs for treating diabetes and its complications.

**Table 1:** Metabolic memory-related drugs

Drugs	Diseases	Animal models	Dose	Cell types	Dose	Mechanisms	Publication time	References
Astragalus polysaccharide	DR	–	–	Human RPE cell line (ARPE-19) and rat primary RPE	12.5 μg/ml, 20 μg/ml, 50 μg/m	ER stress and apoptosis	2020	Peng et al. 2020)
Astragalus polysaccharide	DR	–	–	Human RPE cell line (ARPE-19) and rat primary RPE	12.5 μg/ml, 20 μg/ml, 50 μg/m	mitochondrial dysfunction-induced apoptosis	2019	Liu et al. 2019a)
Dopamine and mdivi-1	DR	C57BL/6 mice + STZ	2 μl of 10 mmol/LL-DOPA every two days	Human RPE cell line and Retinal Müller cells	10 μM	Oxidative stress and mitochondrial dysfunction	2022	Lee et al. 2022)
K9-C-peptide	Retinal vascular leakage, neurodegeneration, pulmonary vascular leakage and fibrosis, glomerular adherens junction disruption, vascular leakage	C57BL/6 mice + STZ	Two times for 4 weeks with 100 mg/kg	Human RPE cell line, endothelial cells	–	ROS generation, TGase activity, and vascular leakage	2023	Jeon et al. 2023)
aPC	DKD	db/m; db/db; TM ^Pro/Pro^mice; TM ^Pro/Pro^x p21 ^−/−^double mutant mice; Human renal biopsy samples	1 mg/kg every other day	HEK-293; Mouse PTC; BUMPT	20 nM	Senescence and fibrosis	2022	Al-Dabet et al. 2022)
Resveratrol	DKD	NOD mice and nondiabetic control group (NOD-C group)	200 mg/(kg/day)	–	–	Inflammation and oxidative stress	2020	Xian et al. 2020)
tBHQ (Nrf2 inducer)	DR	Male Wistar rats + STZ	–	Bovine retinal endothelial cells	15 μM	Oxidative stress (GSH homeostasis)	2014	Mishra et al. 2014)
miR-23b-3p inhibitor	DR	Male Sprague–Dawley (SD) rats + STZ	–	Human RPE cell line	–	Inflammation	2016	Zhao et al. 2016)
Drp1inhibitor and mdivi-1	DR	Male Wistar rats + STZ	–	Endothelial cells isolated from nondiabetic human retina	100 μM	Mitochondrial Dynamics	2022	Mohammad and Kowluru 2022)
Leflunomide (an Mfn2 activator)	DR	–	–	Human retinal endothelial cells	100 μM	Quality control of mitochondria (fusion, mitophagy, formation of new mitochondria)	2023	Kowluru and Alka 2023)
AAV -sirt 7/sirt 7 overexpression-plasmid	DKD	Male Sprague Dawley rats + STZ	–	Human glomerular endothelial cells	–	Inflammation	2022	Li et al. 2022c)
5-Aza	DF	Mice with STZ	–	Human umbilical vein endothelial cells	–	Inflammation and endothelial dysfunction	2021	Zhao et al. 2021)

*ER* endoplasmic reticulum, *RPE* retinal pigment epithelial cells, *STZ* streptozotocin, *aPC* coagulation protease-activated protein C, *ROS* reactive oxygen species, *TGase* transglutaminase, *db/m* C57BLKsJ-db/ + , *db/db* diabetic C57BL/KSJdb, *HEK-293* human embryonic kidney cells, *PTC* primary proximal tubular cells, *BUMPT* Boston University mouse proximal tubular cells, *NOD* nonobese diabetic, *tBHQ* *tert*-butylhydroquinone, *GSH* glutathione, *AAV* adeno-associated virus, *5-Aza* 5-aza-deoxycytidine. “—” denotes unknown

SGI-1027, a highly lipophilic small-molecule inhibitor of DNMT1 based on its quinoline structure, potently inhibits DNA methylation, thereby suppressing senescence, apoptosis, and fibrosis (Sun et al. 2018; Gao et al. 2022; Wang et al. 2019). In DKD, DNMT1 regulates senescence and fibrosis by modulating the DNA methylation status of *p21* (Al-Dabet et al. 2022). Given these findings, we hypothesize that SGI-1027 may hold promise for mitigating hyperglycemia memory and hyperglycemic memory-induced senescence, apoptosis, and fibrosis.

Chaetocin, a small-molecule natural product isolated from Chaetomium fungi, can regulate several mechanisms of metabolic memory, such as apoptosis, oxidative stress, autophagy and immune function (Jiang et al. 2021). *SUV39H1* regulated sustained inflammation in vascular cells that were transiently cultured in high glucose through the modification of H3K9me3 (Villeneuve et al. 2008). Moreover, chaetocin can also decrease histone H3K9me3 levels at the promoter of the *p21 WAF1* gene, which has also been proven to be a hyperglycemic memory-related gene in DKD (Al-Dabet et al. 2022; Lin et al. 2016). Interestingly, *miR-125b*, a key ncRNA that regulates hyperglycemic memory, plays an upstream role in the regulation of inflammatory genes in diabetic mice by downregulating SUV39H1 (Villeneuve et al. 2010; Wang and Chang 2011). These results further support the notion that chaetocin or a miR-125b inhibitor may be effective inhibitors of metabolic memory.

Research on these drugs is currently only at the experimental stage due to safety and other reasons, so their clinical use is still limited. Targeting the site of metabolic processes without interfering with regular metabolic processes is still a challenge. However, understanding the mechanisms of metabolic memory in diabetic complications is benefit in exploring new therapeutic approaches.

## Models of metabolic memory

The concept of metabolic memory was proposed in 2003, with studies involving insulin treatment groups and an average follow-up duration of 6.5 years (Pop-Busui et al. 2009). More recently, Al-Dabet et al. (2022) used SGLT2i for 7.2 ± 0.8 months to manage hyperglycemia and evaluated urinary P21 expression as a marker of persistent tubular damage in DKD. Li et al. (2022c) tested DAPK3 in kidney tissue from DKD patients with poor HbA1c (10.2 ± 3.9) and those with good glycemic control (HbA1c 5.4 ± 0.5). However, crucial details such as the hypoglycemic medications used and the duration of glycemic control were omitted from their study. Hui et al. (2021) divided participants into three groups: a glycemic control group (regular treatment with normal glycemic levels in the past 5 years), a glycemic non-control group (non-regular treatment with poor glycemic control in the past 5 years), and a metabolic memory group (regular treatment and normal glycemic levels in the past year but non-regular treatment with poor glycemic control a year ago). Nevertheless, they also did not describe the hypoglycemic medications used in detail. Given the inherent challenges in controlling variables in clinical research, the majority of studies have resorted to animal and cell models to investigate the mechanisms underlying metabolic memory. Regrettably, there is a lack of consistency in the models of metabolic memory, both in vitro and in vivo. Thus, Table 2 lists different models used in different studies.

**Table 2:** Models of metabolic memory

Diseases	Cell types	Methods for in vitro models	Methods for in vivo models (animal; hypoglycemic medications; days)	References
DKD	Mouse PTCs	24 h for high glucose and 24 h for normal level	Mouse; SGLT2i; 6 weeks	Al-Dabet et al. 2022)
DKD	Human glomerular endothelial cells	3 days for high glucose and 3 days for normal level	Rat; Insulin; 3 weeks	Li et al. 2022c)
DKD	Human PTCs	Derived human PTCs from people with type 2 diabetes and cultured under normal glucose for 4 passages	–	Bansal et al. 2020)
DR	HRECs	2 days for high glucose and 5 days for normal level	Rat; Insulin; 3 months	Zhao et al. 2016)
DR	BRECs	4 days for high glucose and 4 days for normal level	Rat; Insulin; 3 months	Mishra et al. 2014)
DR	HRECs	4 days for high glucose and 4 days for normal level	Rat; Insulin; 4 months	Mohammad and Kowluru 2022)
DCM	AC 16 cells	–	Mouse; Insulin; 4 weeks	Zhan et al. 2023)
Diabetic arterial vasculopathy	Human aortic endothelial cells	1 days for high glucose and 6 days for normal level	Rat; Insulin; 12 weeks	Zhong et al. 2015)
DF	Human aortic endothelial cells	① 1 days for high glucose and 2 days for normal level; ② 1 days for high glucose and 4 days for normal level; ③ 1 days for high glucose and 6 days for normal level	Mouse; Insulin; 2 months	Zhao et al. 2021)

*PTCs* proximal tubular cells, *HRECs* human retinal endothelial cells, *BRECs* bovine retinal endothelial cells. “—”denotes unknown

For in vitro models of metabolic memory, most researchers used high glucose conditions in cultured cells and then changed the glucose concentration to a normal level to simulate intensive treatment in DCCT/EDIC studies. In the majority of studies exploring diabetes and its chronic complications, a hyperglycemic exposure period of 48–72 h is typically considered representative of chronic hyperglycemia with long-term deleterious effects. This holds true for conditions such as DKD, DF, and diabetic cardiomyopathy (Hu et al. 2024; Wang et al. 2024; Li, et al. 2024; Song et al. 2023; Feng et al. 2023). However, it is worth noting that the duration of exposure to high glucose, as well as the periods of exposure to normal glucose, vary significantly across studies examining metabolic memory. In DKD, Al-Dabet et al. (2022) Mouse primary tubular cells were cultured under high-glucose conditions for 24 h and then at normal levels for another 24 h. However, Li et al. (2022c) Human glomerular endothelial cells were exposed to high levels of glucose for 3 days, followed by 3 days under normal conditions. Although these studies cultured cells for different durations, they equally distributed the time to high and normal glucose levels. Zhong et al. (2021) compared different in vitro models of metabolic memory in DF. Interestingly, different models performed similarly, and they used cultured human aortic endothelial cells for 1 day under high glucose and 6 days under normal glucose conditions. Another uncommon method involves deriving human proximal tubular epithelial cells from people with type 2 diabetes and culturing them under normal glucose conditions for 4 passages to establish a metabolic memory model (Bansal et al. 2020). Regrettably, there are no accepted standards for metabolic memory models in vitro*,* and most studies have chosen these models directly. However, for different cell lines, comparing different culture times might be more accurate.

For in vivo models of metabolic memory, the majority of studies have utilized insulin to lower blood glucose levels in diabetic mice or rats. Notably, in 2022, Al-Dabet et al. (2022) reported a novel attempt to employ SGLT2i in the construction of a metabolic memory model. Although they presumably considered the renal benefits of SGLT2i, they did not compare its effectiveness with that of insulin in model establishment. The duration of glycemic control in these studies varied significantly, ranging from a minimum of 3 weeks in rats with DKD treated with insulin to a maximum of 4 months in rats with DR also treated with insulin (Li et al. 2022c; Mohammad and Kowluru 2022). This wide range raises the following question: what is the optimal duration for the construction of in vivo models of metabolic memory? Furthermore, does this duration differ across various diabetic complications?

## Outlook

In this review, we explored the regulatory mechanism of metabolic memory, including inflammation and immunity, oxidative stress and mitochondrial dysfunction, senescence and various kinds of cell death. Then, we discuss the function of metabolic memory in diabetic complications. In addition, we analyzed confirmed and potential metabolic memory-related inhibitors. Finally, we also summarized the in vitro and in vivo models of metabolic memory. In conclusion, metabolic memory might be a vital mechanism in the occurrence and development of various diabetic complications and is a promising therapeutic target for preventing the progression of complications.

There have been extensive studies on the essential mechanisms and regulators of metabolic memory, oxidative stress, mitochondrial dysfunction and apoptosis, which are the most studied and are mainly focused on DR. On the one hand, these traditional mechanisms may be the core mechanisms of metabolic memory and need to be validated more widely for the treatment of different diabetic complications. However, emerging mechanisms, such as senescence, ferroptosis, and pyroptosis, also deserve further exploration. In addition, animal and cellular models of metabolic memory are still controversial. Thus, we speculated that for different cell lines and methods for generating diabetic models, preexperiments may be necessary. Furthermore, numerous key molecules, including *miR-320*, *p21* and *Sod2*, have been identified. However, there are currently no well-recognized markers that can represent metabolic memory in vitro or in vivo, such as ColI and fibronectin for fibrosis, let alone in vitro diagnostic markers used in clinical practice. In addition, small molecule drugs, such as polysaccharide, dopamine and aPC, have also been found to alleviate hyperglycemic memory to mitigate diabetic complications. We also speculated that SGI-1027 and chaetocin may be potential molecular compounds against metabolic memory. Regrettably, these drugs have been limited to animal and cellular models.

In the future, further studies of metabolic memory may start from the following perspectives: (1) develop explicit animal and cellular models of metabolic memory; (2) elucidate the mechanisms of metabolic memory and further uncover more hub molecules that regulate metabolic memory to obtain representative markers; (3) transform small molecule compounds that can be used to regulate metabolic memory in clinical practice; and (4) extensively study the crosstalk between lncRNAs and miRNAs; however, regrettably, pertinent research on the intricate network of lncRNAs and other ncRNAs remains to be conducted.

Although there have been certain studies that have summarized the relationship between metabolic memory and DKD, DR or epigenetic modification, studies on the connection between metabolic memory and chronic complications of DM, along with potential therapeutic drugs, remain scarce. Notably, our review is the first to comprehensively summarize various models of metabolic memory, given that the exact duration and severity of high-glucose toxicity are still elusive. Despite our efforts to comprehensively review the pathogenesis of metabolic memory in diverse chronic complications of DM, we acknowledge certain limitations, including the constraints of our research perspectives. Nevertheless, with further in-depth exploration of metabolic memory in chronic DM complications and elucidation of its underlying mechanisms, we are confident that this study will pave the way for reliable and innovative therapeutic targets that can retard or even arrest the progression of DM and its associated complications. Additionally, we intend to conduct regular systematic summaries in this field of research.

## Data Availability

The data of this study are included within the paper.

## Abbreviations

DM: Diabetes mellitus
DR: Diabetic retinopathy
DKD: Diabetic kidney disease
IDF: International Diabetes Federation
AGE: Advanced glycation end product
mtDNA: Mitochondrial DNA
SGLT2i: Sodium glucose co-transporter 2 inhibitor
DPP4i: Dipeptidyl peptidase 4 inhibitors
GLP-1RAs: Glucagon-like peptide 1 receptor agonists
DCCT: Diabetes Control and Complications Trial
EDIC: Epidemiology of Diabetes Interventions and Complications
UKPDS: United Kingdom Prospective Diabetes Study
ncRNAs: Noncoding RNAs
TXNIP: Thioredoxin-interacting protein
3′ UTR: 3’ Untranslated region
HPTMs: Histone posttranslational modifications
T2DM: Type 2 diabetes mellitus
SUV39H1: Suppressor of variegation 3–9 homolog 1
H3K4me1: Histone H3 lysine 4 monomethylation
lncRNAs: Long noncoding RNAs
miRNAs: MicroRNAs
DF: Diabetic foot
DCM: Diabetic cardiomyopathy
STZ: Streptozotocin
CD36: Cluster of differentiation 36
EndMT: Endothelial-to-mesenchymal transition
VSMC: Vascular smooth muscle cells
ESKD: End-stage kidney disease
GECs: Glomerular endothelial cells
ET-1: Endothelin-1
TECs: Tubular epithelial cells
aPC: Activated protein C
snRNA-seq: Single-nucleus RNA
snATAC-seq: Assay for transposase-accessible chromatin sequencing
RPEs: Retinal pigment epithelial cells
H3K4: Histone 3 lysine 4
DNMT1: DNA methyltransferase 1
DN: Diabetic neuropathy
ER: Endoplasmic reticulum
ROS: Reactive oxygen species
TGase: Transglutaminase
HEK-293: Human embryonic kidney cells
BUMPT: Boston University mouse proximal tubular cells
NOD: Nonobese diabetic
tBHQ: Tert-butylhydroquinone
GSH: Glutathione
AAV: Adeno-associated virus
5-Aza: 5-Aza-deoxycytidine
PTCs: Proximal tubular cells
HRECs: Human retinal endothelial cells
BRECs: Bovine retinal endothelial cells
